# Numerical and Experimental Correlation Between Half-Cell Potential and Steel Mass Loss in Corroded Reinforced Concrete

**DOI:** 10.3390/ma18225238

**Published:** 2025-11-19

**Authors:** Max Lawrence L. Li, Seong-Hoon Kee, Cris Edward F. Monjardin, Kevin Paolo V. Robles

**Affiliations:** 1 School of Civil, Environmental and Geological Engineering, Mapua University, Manila 1102, Philippines; mllli@mymail.mapua.edu.ph (M.L.L.L.); cefmonjardin@mapua.edu.ph (C.E.F.M.); 2 Department of ICT Integrated Ocean Smart Cities Engineering, Dong-A University, Busan 49315, Republic of Korea; shkee@dau.ac.kr

**Keywords:** half-cell potential, steel mass loss, reinforced concrete, corrosion monitoring, COMSOL Multiphysics

## Abstract

Half-cell potential (HCP) measurement is widely applied as a non-destructive technique for assessing corrosion probability, yet its diagnostic capacity remains limited to probabilistic interpretations rather than quantifying the extent of steel mass loss. Conventional HCP measurements can indicate corrosion probability, but not the actual extent of deterioration. The objective of this study is to examine the potential of HCP measurements to indicate actual corrosion severity by numerically simulating HCP values and correlating them with steel mass loss data. Using published experimental datasets, relationships among corrosion current density (J_(_corr_)_), electrical resistivity (ER), HCP, and steel mass loss (m_L_) were established through regression analysis, while COMSOL Multiphysics v6.2 was employed to simulate HCP responses. The simulations revealed increasingly negative HCP values with higher J_(_corr_)_ and conductivity. A second-order polynomial correlation (R^2^ = 0.9999) was obtained between simulated HCP and measured mass loss (0–20%), enabling quantitative interpretation of corrosion severity, demonstrating that HCP can serve as a predictive indicator of corrosion severity. It is demonstrated that the interpretative value of HCP has potential for quantifying corrosion severity to improve monitoring and maintenance strategies.

## 1. Introduction

Concrete is the most widely used material in the construction industry across countries, and is favored for being versatile, durable, and cost-effective in structural infrastructures [1,2,3,4,5]. However, there have been increasing sustainability and environmental concerns that appeal to the industry to proliferate awareness towards the harmful effects of cement production [3,4]. There have been compounding impacts from its manufacturing, use, and demolition, which often result in natural habitats being disrupted, toxic air and water emissions, and a contribution of around seven to eight percent of man-made carbon dioxide (CO_2_) emissions in the world [2]. It becomes crucial for global sustainability to mitigate or increase the safety and durability of reinforced concrete structures that may lessen such pollution, emissions, and resource depletion [5] as steel reinforcement bar (rebar) corrosion is among the main causes of structural damage, leading to degradation such as cracking, spalling, reduced bond strength, and compromised load-bearing capacity or structural stability [5,6]. It becomes important, then, that early detection and monitoring methods of corrosion are developed to proactively manage and extend the longevity of infrastructures. Corrosion is hardly visible, or not visible at all, especially in its early stages on the surface of concrete, and to that effect, non-destructive testing (NDT) methods have become widely adopted for early corrosion detection as it is allows for assessing the presence or extent of corrosion without damaging the concrete, and due to their simplicity and cost effectiveness, for the timely detection of corrosion risk [5,7,8,9,10].

Globally, the costs attributed to the damage from corrosion roughly reach USD 2.5 trillion, which represents around 3.4% of the global gross domestic product (GDP), as such, corrosion management practices may roughly save up to USD 375 to 875 billion annually [11,12,13,14,15,16]. These costs do not yet include those indirectly related to corrosion, such as the ecological impacts, as all the forests on Earth would not even offset half of the damage caused by the CO_2_ emissions from concrete consumption and production [3]. The damaging impacts caused by its production, utilization, and demolition deplete natural resources, disrupt natural habitats, release toxins to the air and water in its surroundings without proper handling, and contribute to the CO_2_ emissions in the world [2,3,4,12]. According to NACE International, should corrosion mitigation techniques be enforced, the industry would be able to save around 15 to 35% of the total cost due to corrosion in the world, preventing possible catastrophic damages and extending the life cycle of concrete structures. Corrosion on rebars is typically attributed to environmental factors such as chloride ion ingress, moisture exposure, and carbonation [14,15,17,18,19,20,21,22,23]. These environmental factors affect the integrity of the material as it corrodes the reinforcement [9,16].

Corrosion is an electrochemical process that happens when a metal surface interacts with its environment, subsequently resulting in corrosion products, e.g., a metal reacts to a change in pH level, carbonation, oxidation, or chloride penetration, which then results in metal oxides or hydroxides [24,25,26,27,28,29]. Rebars in reinforced concrete structures are typically protected by a passive layer-like film, whose stability, if affected by environmental factors like a decrease in its pH level or exposure to heavy ion concentrations, can become degraded and lead to corrosion [27,30,31]. The corrosion of the rebar in the concrete starts with electrochemical half-reactions; anodic regions form at the areas where steel oxidizes and releases electrons [29], as shown in the following equation:

(1) $$Fe\to {Fe}^{2+}+{2e}^{-}$$

while cathodic regions form in areas where those electrons are consumed through a reduction reaction, as shown in the following equation [13,32]:

(2) $$O_{2}+{2H}_{2}O+{4e}^{-}\to {4OH}^{-}$$

The electrolyte, which is usually the concrete pore solution, that is, the alkaline liquid found in the pores of hardened concrete [33], allows for ionic movement and corrosion reaction [29,34]. Typically, in the high alkaline environment of concrete structures, at a pH level of 12–13, the embedded rebar forms a protective film, primarily made of iron oxides and hydroxides, that keeps it from corroding [35]. However, once that film breaks down due to exposure to harsh environments or agents, such as an ingress of chloride ions (chloride penetration) and carbon dioxide (carbonation), the rebar starts to undergo a corrosion reaction [13,29,32]. Any presence of moisture further accelerates the corrosion process by aiding ion mobility and degrading the protective film more [27]. When these half-reactions happen simultaneously at different regions of the steel surface, corrosion cells are formed. This system has a natural electrical potential, where a high-impedance voltmeter can measure the open-circuit potential difference between the rebar and reference electrode [36,37].

**Figure 1:**
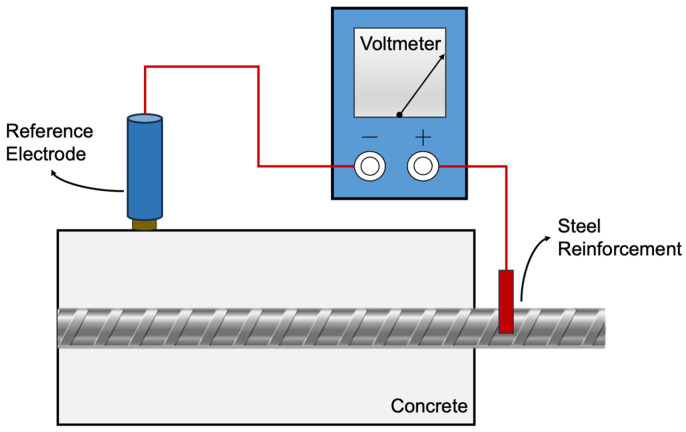
Redrawn half-cell potential measurement from De Carufel [47].

Numerous studies are conducted that focus on evaluating aspects of corrosion related to electrochemistry, such as polarization resistance, corrosion rate, and potential [38,39,40,41,42,43]. Among various NDT techniques, half-cell potential (HCP) measurement has become one of the most widely used field methods for corrosion monitoring [44,45]. HCP assesses the likelihood of corrosion in reinforced concrete structures by evaluating the electrical potential difference between the embedded rebar and a standard reference electrode placed on the surface of the concrete specimen being tested [9,16,24]. There are usually three parts in HCP measurement setups: the rebar as the working electrode, the reference electrode (commonly a copper/copper sulfate electrode or CSE, or a silver/silver chloride electrode or Ag/AgCl), which provides a stable and known potential to be used as comparison, and a high-impedance voltmeter, the conducting wire of which connects to the rebar on one end and another to the reference electrode for the creation of an electrical circuit to measure the potential difference between the rebar and the reference electrode in voltage (see Figure 1) [46].

By establishing an electric circuit between an embedded steel rebar and a stable reference electrode in contact with the concrete surface, one can record the potential difference that indicates the rebar’s electrochemical state [25,27]. Proper electrical continuity, low-resistance contact, and consideration of concrete resistivity are prerequisites for accurate readings [25,28,48]. The rebar is connected to the reference electrode via the high-impedance voltmeter, establishing an electrical circuit, of which measurements would be systematically taken at various points across the surface of the concrete specimen to map the potential variations that serve as indicators of corrosion activity [5,7,30]. The HCP readings serve as an indicator of the probability of corrosion, as they do not directly measure the corrosion rate or nature [49]. The HCP measurements are interpreted using established thresholds, such as the American Society for Testing and Materials (ASTM) C876 [50]. Per ASTM C876 (see Table 1); a potential more negative than −350 mV (vs. Cu/CuSO_4_) is statistically associated with a >90% probability of active corrosion, while values more positive than −200 mV suggest a <10% likelihood [50]. Intermediate values, between −200 mV and −350 mV, necessitate further assessment [51].

HCP measurement has been extensively validated as a diagnostic tool in both laboratory and field environments. It is one of the most commonly used NDT techniques, favored for its simplicity, cost effectiveness, and non-destructive nature [25,33,37,52]. HCP measurement is suitable for in situ evaluations and corrosion risk identification across various locations in a structure due to it requiring only minimal equipment to perform, and further optimizes budget and resource allocation in maintenance works due to its capability to identify zones of higher vulnerability and corrosion risk [5,25,33,37]. Numerous studies confirm its feasibility in early detection across various exposure conditions, including coastal, industrial, and structural contexts [8,24,25,35,37,38,39,40,51]. By mapping out potential differences across concrete structures, its variations in electrochemical activity help determine the most vulnerable locations of the steel reinforcement structure [7,9,16,24,31,39,41,42,43,44,45,51,53,54]. Other studies caution against sole reliance on HCP due to its sensitivity to moisture variations, thermal changes, carbonation, and heterogeneities within the concrete matrix [9,36,41,46,47,55,56,57]. NDT methods, such as HCP measurement, have been shown to necessitate a level of understanding of the various parameters, e.g., moisture content of concrete, its surrounding temperatures, concrete composition, steel type, chloride content, and other external factors, to account for their effect on the measurement and assessment of their readings on corrosion [9,22,39,48,50,58,59,60]. This study, however, focuses on quantifying the direct relationship between HCP and steel mass loss through combined numerical analysis and experimental investigation.

However, the interpretation of HCP remains probabilistic, indicating only the likelihood—not the extent or severity—of corrosion damage [49]. This is a significant limitation in practice because structural management decisions such as repair prioritization or remaining service life estimation require quantitative indicators like steel mass loss rather than probability alone [58,61].

Another key gap in the literature is the limited integration of numerical modeling with HCP measurements [62]. While some computational studies have explored the simulation of corrosion processes, very few have focused on directly simulating HCP response and correlating it with corrosion severity parameters. HCP is fundamentally governed by electrochemical principles, and with appropriate modeling of corrosion current density, electrical resistivity, and boundary conditions, numerical methods can provide deeper insights into the physical mechanisms underlying measured potentials [58,61]. However, systematic studies linking simulated HCP values with experimentally derived steel mass loss remain scarce, leaving a critical gap between theoretical understanding and practical application. Few studies have quantified the direct relationship of HCP to actual steel mass loss using numerical simulation. Existing works typically treat HCP as a qualitative indicator, without integrating it into a predictive model validated against experimental data. This study addresses that gap by developing a numerical–experimental correlation framework linking simulated HCP values to measured mass loss, thereby transforming HCP into a quantitative diagnostic tool for corrosion severity.

That is why this study aims to investigate the potential of HCP measurements to detect the actual steel mass loss in corroded concrete and contribute to the lack in numerical simulations for HCP measurements. Specifically, the objectives of this study are as follows: (1) to establish empirical relationships among corrosion current density, electrical resistivity, and steel mass loss using published experimental datasets; (2) to simulate the electrochemical potential field in reinforced concrete using the Electric Currents physics interface in COMSOL Multiphysics v6.2 (COMSOL AB, Stockholm, Sweden) to replicate HCP measurements under varying corrosion conditions; (3) to develop a predictive correlation between simulated HCP values and steel mass loss, enabling a shift from probabilistic corrosion assessment toward a quantitative evaluation framework; and (4) to validate the numerical findings experimentally using HCP measurements taken with a PROCEQ Profometer (PROCEQ, Zurich, Switzerland) at different stages of accelerated corrosion.

By bridging numerical modeling and experimental measurements, this study demonstrates the feasibility of quantifying corrosion severity based on HCP readings. This approach can enhance the utility of HCP as a practical NDT tool for structural condition assessment, support data-driven maintenance planning, and contribute to the sustainable management of reinforced concrete infrastructure.

## 2. Numerical Simulation

### 2.1. Principle Behind the Simulation

The numerical simulation in this study was made using the Electric Currents physics interface under the AC/DC Module of COMSOL Multiphysics v6.2, and is grounded on the fundamental principles of Ohm’s Law, as expressed in the equation below:

(3) $$J=\sigma E+J_{e}=-\sigma \nabla V+J_{e}$$

where σ is the electrical conductivity, E is the electric field, which equates to −∇V, the gradient of electric potential, and $J_{e}$ is any externally applied current density (e.g., current injection or corrosion current), and on the governing equation of charge conservation, or equation of continuity, as expressed below:

(4) $$\nabla \cdot J=Q_{j}$$

where J is the current density vector and $Q_{j}$ is the current source term [63]. The Electric Currents interface under the AC/DC module in COMSOL Multiphysics v6.2 mainly uses Ohm’s Law to simulate and analyze electric fields, currents, and potential distribution in materials that are conductive [63]. Ohm’s Law describes the relationship between voltage, current, and resistance. According to this law, the voltage measured in an HCP measurement test is affected by the current density, that is, corrosion current density, and the resistance of a medium, that is, electrical resistivity. In this context, this law governs the phenomenon of how HCP measurements are taken. The continuity equation, on the other hand, expresses how electric charge is conserved, such that any current that flows from a material must have a source of origin, either inside the material or from accumulated charge over time [63]. In a steady-state simulation such as the one used in this study, accumulation is considered to be zero and the equation ensures that the spread of current in the material is balanced with the current source term being defined and flowing realistically [63]. The electric potential distribution (V) is then solved using the generalized continuity equation in conductive media, where Ohm’s Law was integrated into the continuity equation as expressed in the following equation:

(5) $$\nabla \cdot (-\sigma \nabla V+J_{e})=Q_{j}$$

The resulting potential field across the concrete was analyzed using point evaluations along the rebar to mimic the practical measurement of HCP.

Although corrosion is inherently three-dimensional and time-dependent, a 2D steady-state model was employed to simplify the domain while accurately capturing the electrochemical potential distribution around the rebar cross-section under constant environmental conditions. The reference potential (0 V) represents the surface electrode potential, and the floating potential at the rebar boundary allows for direct determination of HCP values relative to this node. Mesh convergence was verified by refining until HCP variation between successive refinements was less than 1%, confirming mesh independence. The model accounts solely for electrochemical behavior; mechanical stresses and strain-induced cracking effects are beyond the present scope and may be addressed in future multiphysics coupling work. Environmental effects such as temperature and moisture gradients were not explicitly modeled; the analysis assumes isothermal, fully saturated conditions of a controlled setup. Time-dependent moisture transport, carbonation, and cracking are outside the present study’s scope and are likewise recommended to be considered in future electrochemical transport mechanical coupling studies.

### 2.2. Model Geometry and Input Parameters

**Figure 2:**
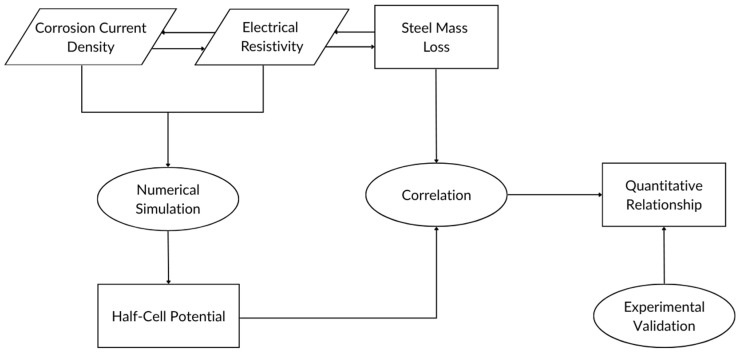
Graphical overview of this study’s workflow showing the interrelation between numerical simulation, correlation development, and experimental testing.

**Figure 3:**
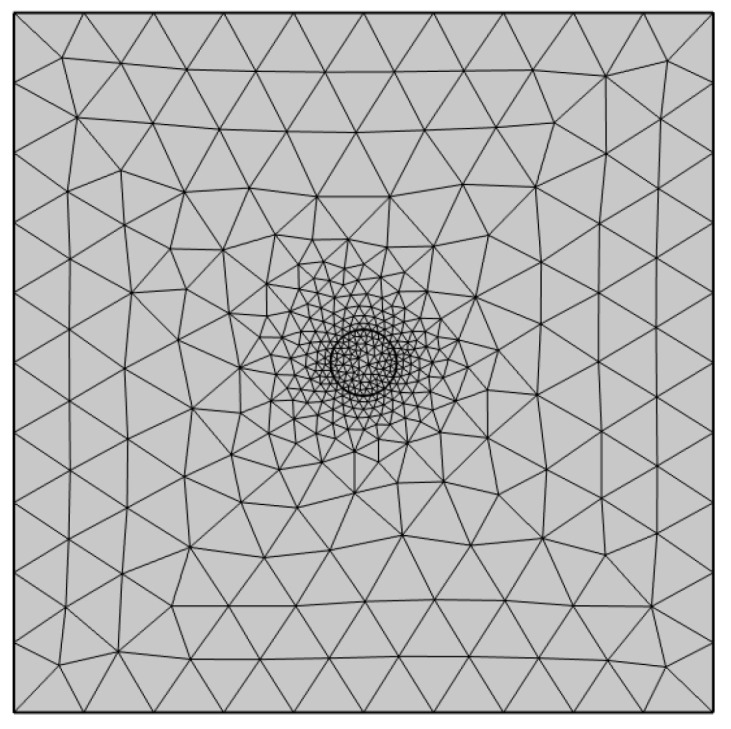
Mesh refinement applied to 2D finite element model (200 × 200 mm concrete cube with a 19 mm rebar at the center).

Figure 2 shows the procedural flow for creating the HCP measurement simulation in COMSOL Multiphysics v6.2, where a 2D finite element model was developed to simulate the HCP distribution in reinforced concrete blocks and analyze it in relation to corrosion. The simulation model is designed to replicate the experimental setup, using a 200 mm (length) × 200 mm (width) concrete cube with a 19 mm centrally embedded steel rebar [5]. Mesh refinement was applied with a free triangular mesh, applying finer elements around the steel-concrete interface to improve solution accuracy near the current injection sites (see Figure 3). The minimum element size was set to 0.025 mm and the maximum element size was set to 7.4 mm (predefined Finer mesh) around the rebar boundary, and a minimum of 0.4 mm and a maximum of 20 mm (predefined Coarse mesh) in the concrete bar, ensuring convergence of potential gradients. It should be noted that the numerical simulation assumed steady-state conditions, and all materials was considered homogeneous and isotropic.

To model corrosion, Normal Current Density, a cathodic boundary condition, was applied along the rebar perimeter using the parameter −J_(_corr_)_, where the negative value would simulate anodic corrosion of steel and mimic the behavior of electrons leaving the domain of the rebar at that boundary. Corrosion current density (J_(_corr_)_) values were made sweepable and assigned based on the values gathered from the regression analysis of the relationship between electrical resistivity and corrosion current density, as discussed below. The reference electrode was simulated by assigning a 0 V Electric Potential condition to a designated boundary on the concrete surface, enabling the rest of the electric potential field to be evaluated relative to this point. Furthermore, a Floating Potential condition was applied on the boundary of the rebar to allow the simulation to solve for the potential at the rebar, also relative to the reference electrode; though the electric potential of the rebar may already be probed without this condition, an assigned Floating Potential allows for simplicity and efficiency in determining the HCP values at the rebar. Boundary conditions were applied as follows: the top surface of the concrete domain was grounded (0 V reference), while the rebar boundary was set as a floating potential, allowing the potential to equilibrate based on the corrosion current density distribution. Mesh convergence was tested by refining element sizes until potential variation between successive meshes was less than 1%, and the final model used approximately 45,000 quadrilateral elements, with a minimum size of 1 mm near the steel–concrete interface. Material properties included electrical conductivity of 0.01 S/m to 1 S/m and relative permittivity of 1 for concrete, and conductivity of 6.9 × 10^6^ S/m for steel.

The relationship between corrosion current density (*J*_(_corr_)_) measured in A/m^2^ and electrical conductivity (σ) measured in S/m was derived from a published study [64], which established an empirical sixth-order polynomial correlation based on experimental datasets covering *J*_(_corr_)_ values from 0.001 A/m^2^ to 0.8 A/m^2^. The equation, originally formulated to represent the nonlinear dependence of pore-solution conductivity on corrosion activity, was as expressed in Equation (6). Table 2 presents the input data used in this simulation.

(6) $$\sigma =-6.9778\cdot {J_{\left(corr\right)}}^{6}+14.408\cdot {J_{\left(corr\right)}}^{5}-9.8241\cdot {J_{\left(corr\right)}}^{4}+2.3899\cdot {J_{\left(corr\right)}}^{3}-0.1137\;\cdot {J_{\left(corr\right)}}^{2}+0.0182\cdot J_{\left(corr\right)}+0.0033\;\;\;\;\;\;$$

### 2.3. Half-Cell Potential and Steel Mass Loss

From the derived electrical conductivity (σ) values, the corresponding electrical resistivity (ER) values were computed, since resistivity is the reciprocal of conductivity, as expressed by the following equation:

(7) $$ER=\frac{1}{\sigma }$$

These ER values are crucial for the analysis of this study as they act as the hinge for relating the HCP values simulated with the m_L_ [65,66,67]. Given the equivalent ER values corresponding to the J_(_corr_)_ values, the relative electrical resistivity (*r_e_*) values were then calculated by normalizing each ER value to the first data point, following the equation below:

(8) $$r_{e}=\frac{{ER}_{at\;specific\;Jcorr}}{{ER}_{initial}}$$

To establish the relationship between electrical resistivity and actual corrosion damage, this study adopted data from a previously published experimental investigation [5] that examined the relationship between *r_e_* and steel mass loss (m_L_) in reinforced concrete specimens. The referenced study included three concrete mixes—Mix 1, Mix 2, and Mix 3—with distinct mechanical and physical characteristics to account for differences in strength, porosity, and permeability, as shown in Table 3. Mix 1 had a design strength of 18 MPa with a water-to-cement (w/c) ratio of 0.585, Mix 2 had 24 MPa with a w/c ratio of 0.507, and Mix 3 had 40 MPa with a w/c ratio of 0.346. Each mix was composed of varying proportions of water, cement, aggregates, and air-entraining agents to capture the influence of concrete quality on the corrosion process.

**Figure 4:**
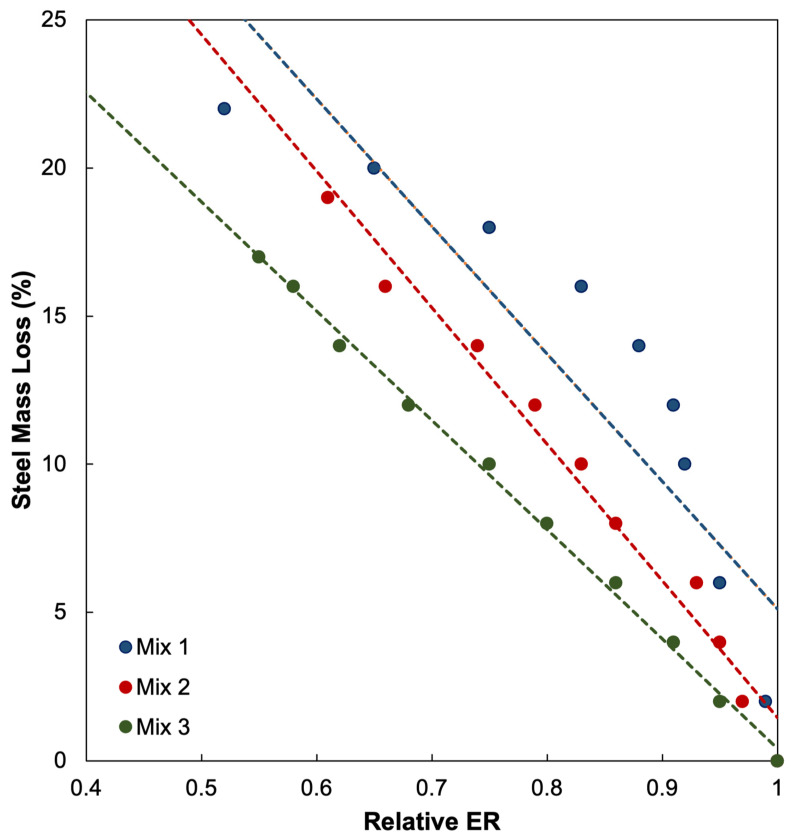
The relationship of relative electrical resistivity (*r_e_*) and steel mass loss (m_L_) values of Mix 1, Mix 2, and Mix 3.

Figure 4 and Table 4 present the regression relationships between *r_e_* and m_L_ for the three concrete mixes, where m_L_ was expressed as a linear function of *r_e_*. These equations allowed for the estimation of mass loss corresponding to specific resistivity ratios. The observed scatter in HCP readings, particularly at intermediate corrosion levels, can be attributed to several interacting parameters within the concrete specimens. The uncontrolled environmental and material factors, such as varying local moisture content, microcrack formation, surface carbonation, ambient temperature, and concrete heterogeneity among other factors, are difficult to replicate precisely across specimens and can influence the ionic conductivity, electrochemical potential, electrical resistivity, or corrosion current density. Consequently, the dispersion observed in the experimental data reflects the variability inherent in experimental setups unlike the idealized and controlled conditions assumed in the numerical simulation. Notably, the lower R^2^ value observed for Mix 1 likely results from its higher water–cement ratio and increased porosity, which introduce greater variability in electrical resistivity and HCP measurements. Despite this, the overall correlation trend remains consistent across all mixes. The relative ER values derived in this numerical study were substituted into these regression equations to calculate the equivalent m_L_ values for each mix.

Afterward, in order to correlate the simulated HCP values with the corresponding mass loss, the researchers related the computed m_L_ values to the σ (conductivity) and J_(_corr_)_ parameters used in the COMSOL simulation. Because electrical conductivity is the inverse of resistivity, this approach effectively bridges the experimental resistivity-based findings with the numerical conductivity-based simulations. As a result, a direct relationship between the simulated HCP and the corresponding m_L_ was established, enabling a quantitative interpretation of corrosion severity based on the numerically simulated electrochemical potential field.

Since the J_(_corr_)_ and σ inputs were identical across all mixes, the simulated HCP values remained constant for each set, while the variation was observed only in the m_L_ values derived from the regression analysis. To ensure comparability among the three mixes, the dataset was restricted to a relative ER value of 0.52, corresponding to the lowest common ER range reported in the reference study [5]. The resulting data were then analyzed through regression to assess the predictive capability of HCP for quantifying actual corrosion in reinforced concrete.

## 3. Experimental Study

### 3.1. Preparation of Specimens

The experiment involved 36 concrete cube specimens, measuring 200 mm in width and 200 mm in length, and 235 mm long rebars, measuring 19 mm in diameter. Of the 235 mm long rebar, 135 mm was embedded into the center of the specimen; as such, the working area of the rebar for the steel corrosion was 4176 mm^2^. The specimens were constructed using wooden forms that were 20 mm thick; the rebar was horizontally placed through a hole in one side of the wooden form. The rebar used conformed to the KS D 3504 standard [5], which typically comprises approximately 0.24% carbon (C), 0.80–1.60% manganese (Mn), 0.30% silicon (Si), and trace amounts of phosphorus (P) and sulfur (S), all within the standard permissible limits. The non-contact length of the rebar was coated with two layers of epoxy and one layer of urethane; the urethane-coated area was then wrapped with a polytetrafluoroethylene (PTFE) tape, and a 100 mm polyvinyl (PVC) pip was added for further protection to prevent corrosion.

The mixture was composed of 168 kg/m^3^ of water, 287 kg/m^3^ of Portland cement type I, 898 kg/m^3^ of gravel, 957 kg/m^3^ of sand, and 2.58 kg/m^3^ of high-performance air-entraining agent (see Table 5). The concrete mix composition was also used to make 36 cylindrical concrete specimens that were 100 mm in diameter and 200 mm in length to account for the material properties of the concrete, e.g., material strength and saturation curves; the results were a design strength of 18 MPa and a water-to-cement (w/c) ratio of 0.585, as seen in Table 5. The specimens were stored for post-casting and curing in a room that was at a constant 20 ± 3 °C humidity to remain air-dry before saturation.

### 3.2. Accelerated Corrosion and Actual Steel Mass Loss

**Figure 5:**
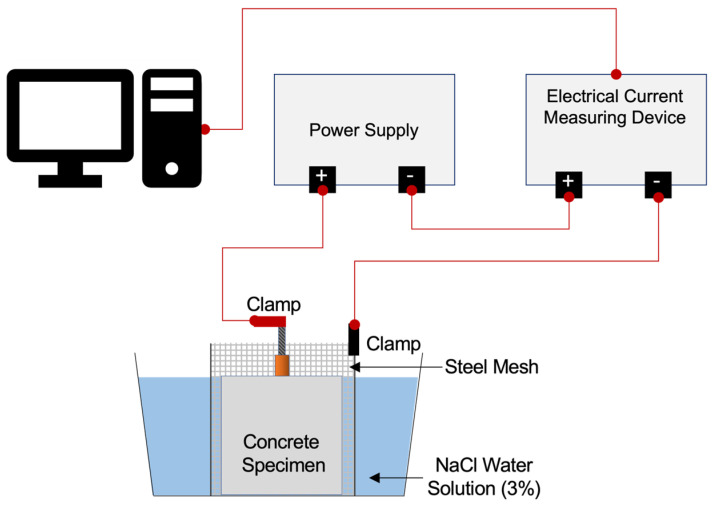
Schematic diagram of the accelerated corrosion.

The concrete specimens were treated using the impressed current technique to exhibit various levels of steel corrosion [68,69,70,71,72]. The specimens were submerged in a 3.0% NaCl solution for seven days to ensure full saturation, with each cube positioned so that its top surface was entirely submerged within the NaCl solution. A stainless-steel mesh (SUS 316) was wrapped around the side surfaces of the concrete specimen to act as the cathode, to which the embedded rebars served as the anodes. As shown in Figure 5, a constant voltage input (maximum applied current = 1.05 A) was delivered to the rebar from a DC power source (ODA Programmable DC Power Supply–OPE-DI Series, Ainuo, Incheon, Republic of Korea), where its positive terminal was connected to the rebar, while its negative terminal was linked to an electrical current measuring device (KEYSIGHT Truevolt Digit Multimeter, KEYSIGHT, Santa Rosa, CA, USA). The corrosion process was monitored in real time using a computer via LabVIEW 2016 software and the corrosion levels of the 19 mm rebars were categorized based on theoretical steel mass loss: 0%, 5%, 10%, and 20%. The specimens showed cracks on their surfaces after being subject to the impressed current technique. An increase in the width of the cracks was also observed as the corrosion levels increased and the corrosion product surfaced as rebar corrosion advanced.

**Figure 6:**
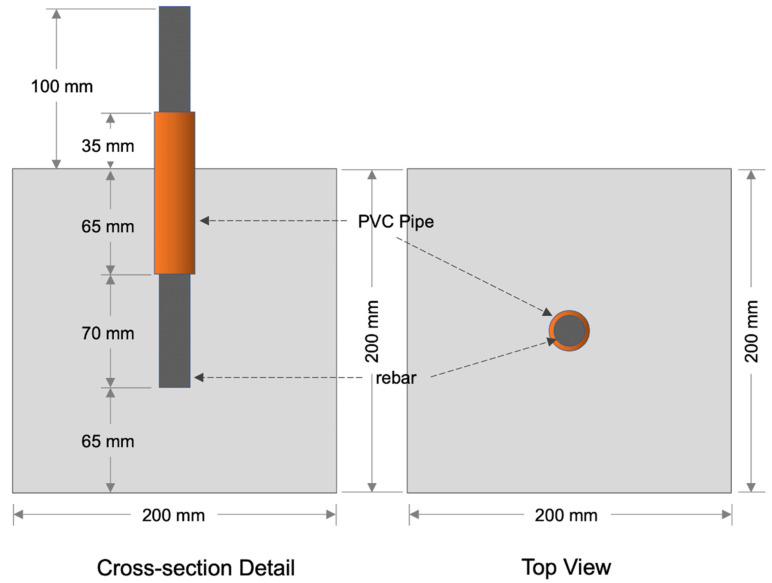
Schematic diagram of the concrete specimens used in this experiment.

The actual steel mass loss was quantified following the standard procedure outlined in ASTM G1—“Standard Practice for Preparing, Cleaning, and Evaluating Corrosion Test Specimens.” After removing the corroded rebars from the concrete cubes, they were first subjected to ultrasonic cleaning and subsequently immersed in a sodium hydroxide (NaOH) solution to dissolve and remove corrosion products. Any remaining rust that was not eliminated through this chemical cleaning was further removed through sandblasting, wherein a high-pressure air jet was used to strip off the residual rust layer and expose the sound steel surface. As illustrated in Figure 6, the corrosion zone of the rebar specimens in this study was restricted to a 70 mm segment, while the rest of the embedded steel length was protected from corrosion through epoxy coating and PVC sleeving.

The mass loss of steel was determined based on Archimedes’ principle of buoyancy. To calculate the volume of the steel corresponding to the corroded region, the rebar was submerged in water up to a 70 mm corrosion length. The volume of water displaced was taken as the volume of the steel specimen. Using the known density of steel, the corresponding mass of the steel was then calculated. Finally, the steel mass loss ratio (mL), representing the corrosion level in this study, was obtained by normalizing the measured mass loss from the corroded portion to the initial mass of the uncorroded rebar segment of the same length. Mathematically, it can be expressed as Equation (9), where *m_air_* is mass of a rebar measured in air, *m_w_* (L) is mass of a rebar that was partially immersed in water at the height of the working area (70 mm from the tip of the rebar), *m*_0_ is mass of the working area of uncorroded rebars before accelerated corrosion test, and ρ_s_ and ρ_w_ are mass densities of steel and water, respectively.

(9) $$m_{L}=\left[1-\frac{{(m}_{air}-m_{w}(L))\rho_{s}}{m_{o}\rho_{w}}\right]\times 100\;[\%]$$

The steel mass loss in this study was determined using the buoyancy (Archimedes displacement) method following ultrasonic and chemical cleaning per ASTM G1. This approach allows the mass loss to be isolated specifically to the 70 mm active corrosion region, avoiding the uncertainty that may arise when weighing the entire bar including uncorroded segments. Although gravimetric weighing before and after corrosion is commonly used in the literature, both methods rely on the same cleaning procedure, and differences primarily originate from water meniscus effects, temperature-dependent density, and balance resolution. In the present work, repeated buoyancy measurements demonstrated highly consistent results, with no discernible fluctuation beyond normal measurement tolerance. The authors acknowledge that a direct comparison between buoyancy-derived and gravimetric measurements was not included; however, the high repeatability observed supports the suitability of the buoyancy approach for localized mass loss quantification. Future studies will incorporate paired gravimetric validation to statistically assess method agreement and further enhance measurement confidence.

### 3.3. Half-Cell Potential Measurements

**Figure 7:**
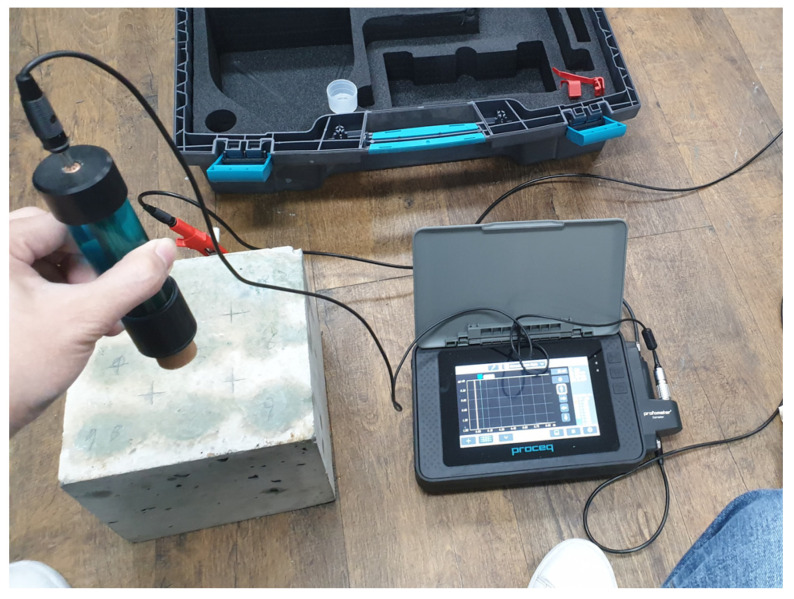
Experimental setup for half-cell potential (HCP) measurement showing a PROCEQ Profometer Corrosion GP meter, a Cu/CuSO_4_ reference electrode, and a specimen under test connected through a high-impedance voltmeter.

The HCP measurements were performed in accordance with ASTM C876 to evaluate the electrochemical activity of the embedded steel reinforcement at different corrosion levels [50]. A PROCEQ Profometer with an integrated copper/copper sulfate electrode (CSE) and high-impedance voltmeter was used to obtain all measurements, as shown in Figure 7. Prior to testing, the top smooth surface of each concrete specimen was lightly cleaned and moistened to ensure good electrical contact between the reference electrode and the concrete.

The reference electrode was positioned at a single measurement point located at the center of the 200 × 200 mm top surface of the specimen, directly above the embedded rebar. The electrical connection to the rebar was made through a lead wire attached to its exposed end. Once contact was established, the Profometer recorded the open-circuit potential difference between the rebar and the reference electrode. The reading was allowed to stabilize before recording, and each measurement was repeated five times to obtain an average value and minimize random fluctuations. The measurements are carried out at different corrosion stages.

For each specimen, half-cell potential (HCP) was measured five times at the same point. Before recording each value, the reading was allowed to stabilize, meaning the voltage drift was less than 2 mV over a 30 s period. The average of the five stabilized readings was used as the representative HCP value for that specimen. Across all specimens, the variation among repeated readings was consistently small (≤5 mV), indicating stable measurements.

## 4. Results and Discussion

### 4.1. Distribution of Electrical Potential in Reinforced Concrete

**Figure 8:**
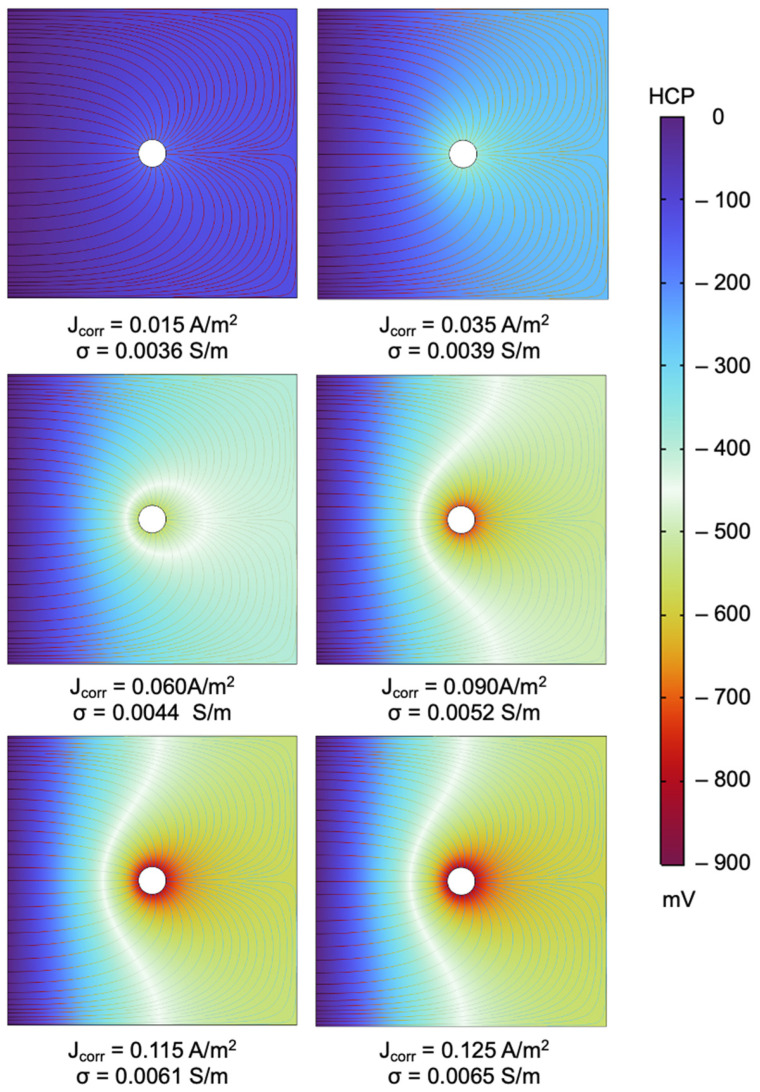
Electric potential field distribution generated in COMSOL across different values of Jcorr and sigma.

Figure 8 illustrates the simulated distribution of electric potential within the reinforced concrete domain, where the circular region at the center represents the embedded steel rebar, generated by the COMSOL simulation. The potential field ranges from approximately 0 mV (dark purple zone) at the left boundary, corresponding to the applied reference potential, to around −793 mV (dark red zone) concentrated at the surface of the rebar. The contour lines highlight the gradient of potential radiating outward from the rebar, which displays the transfer of charge in an electrochemical corrosion system. The gradient also shows the anodic activity of the corroding rebar by reflecting how the most negative potentials are concentrated at the steel–concrete interface, reflecting the outward spread of the potentials through ionic transport during anodic dissolution, which generates electrons at the surface of the rebar.

As the J_(_corr_)_ increases from 0.015 A/m^2^ to 0.125 A/m^2^, accompanied by higher σ, the HCP values also become increasingly more negative and the red zone of the electric potential field distribution is observed to further expand. This pattern shows that the higher corrosion activity which surfaces around the rebar leads to more negative steel potentials that extend into the domain of the concrete. At the lowest corrosion current density, shown in Figure 8 at J_(_corr_)_ = 0.015 A/m^2^ and σ = 0.0036 S/m, the potential distribution is relatively uniform with a limited spread of negative potentials around the rebar, as seen in the slightly lighter hue near the rebar, the gradient of which reflects minimal corrosion activity. At J_(_corr_)_ = 0.035 A/m^2^ and σ = 0.0039 S/m, however, the gradients become more visible, with the colors expanding to yellow and a little light green around the rebar, depicting the development of stronger corrosion activity. On the other hand, J_(_corr_)_ = 0.060 A/m^2^ and σ = 0.0044 S/m shows a distinct negative potential zone surrounding the rebar with more visible yellow contours, which only intensified into a red color at J_(_corr_)_ = 0.09 A/m^2^ and σ = 0.0052 S/m. At J_(_corr_)_ = 0.115 A/m^2^ and σ = 0.0061 S/m and at J_(_corr_)_ = 0.125 A/m^2^ and σ = 0.0065 S/m most pronounced gradients with a dark red color are observed, as the electric potential field distribution become indicative of more aggressive corrosion activity that influences a wider region of the concrete. The nonlinear potential trend corresponds to Tafel behavior, where increasing corrosion current densities shift the electrode potential logarithmically. The observed potential saturation at high J_(_corr_)_ is typical of polarization limits in anodic reaction zones. The generated potential contours suggest the potential of numerical simulations to detect localized corrosion and map vulnerabilities to supplement insights into the spatial distribution of corrosion activity.

**Figure 9:**
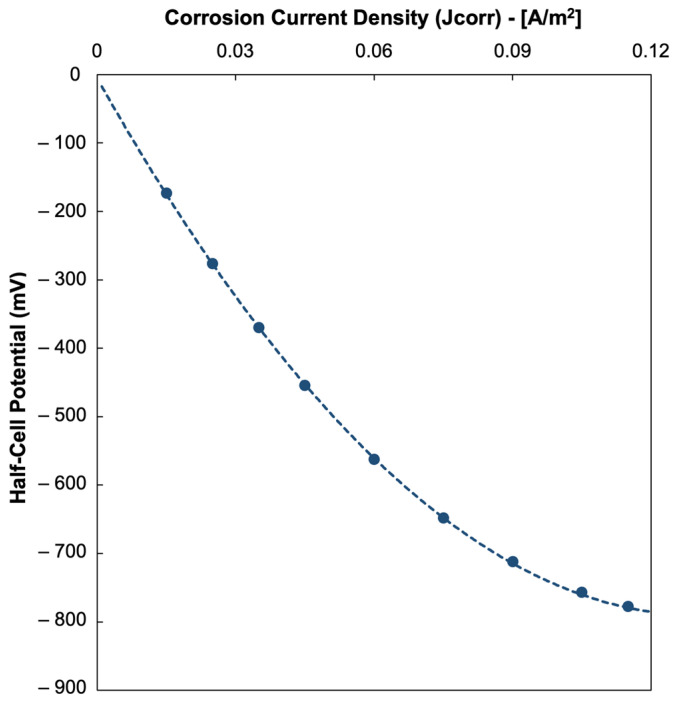
Relationship of simulated half-cell potential (HCP) vs. corrosion current density (J_(_corr_)_).

**Figure 10:**
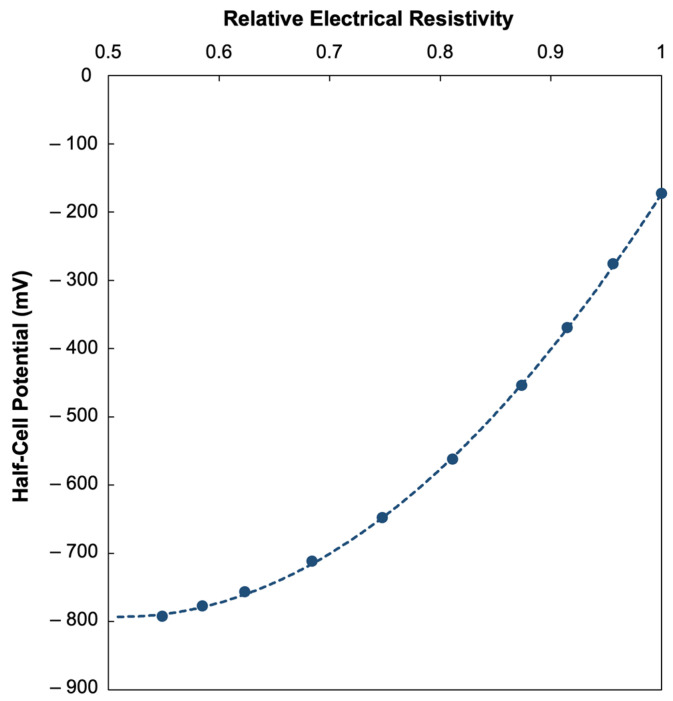
Relationship of simulated half-cell potential (HCP) vs. relative electrical resistivity (*r_e_*).

The relationship between the simulated HCP and J_(_corr_)_, as seen in Figure 9, demonstrate increasingly negative HCP values for higher corrosion current densities. This trend corresponds to the electrochemical principles that higher corrosion current densities are associated with more anodic processes at the steel surface, while increased electrical conductivity of the pore-solution facilitates ionic transport, both of which generate more negative potentials being detected from the corroding rebar [13,32,36]. The steep initial slope of the curve suggests that small increases in J_(_corr_)_ at lower values produce a sharper decline in HCP, which eventually flattens as J_(_corr_)_ increases further, implying that there is a saturation effect in which additional increases in corrosion activity have a diminishing change to the HCP values. Similarly, the relationship between HCP and *r_e_*, shown in Figure 10, reflects how an increase in ER corresponds to less negative HCP values. This trend may be explained by the restriction of ionic transport and suppression of corrosion currents experienced by more dense and less conductive concrete due to the increase in electrical resistivity. On the other hand, low resistivity concrete allows for higher corrosion currents that lead to more negative HCP values. These results highlight that HCP measurements cannot be interpreted in isolation as, though negative HCP values strongly relate with high J_(_corr_)_ and corrosion activity, the effect of ER must also be considered to avoid possible misinterpretations of the data.

### 4.2. Relationship Between Half-Cell Potential of Concrete and Steel Mass Loss

**Figure 11:**
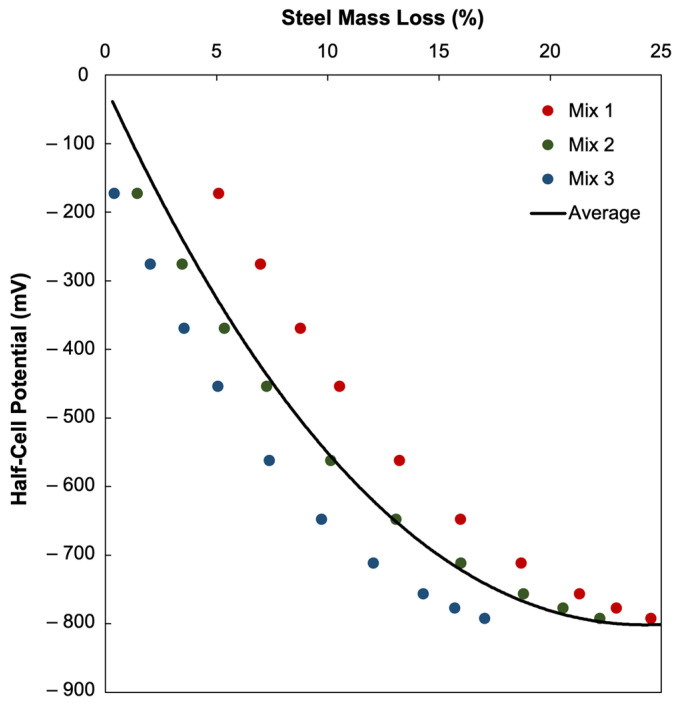
The average relationship of half-cell potential (HCP) and steel mass loss (m_L_) of Mix 1, Mix 2, and Mix 3.

Figure 11 shows the correlation between the simulated HCP values and m_L_ corresponding to the corrosion in an embedded rebar across three different concrete mixes (Mix 1, Mix 2, and Mix 3). The m_L_ values were derived from their relationship with *r_e_* reported in the published studies [5], as detailed in Section 2.2. This correlation expresses HCP values as a function of m_L_, thereby linking the HCP measurements to the actual corrosion severity through the m_L_ of rebars.

For all three mixes, the HCP values exhibit an increasingly negative trend as m_L_ progresses. For Mix 1, HCP values declined from approximately −173.49 mV at around 5.114% m_L_ to nearly −793.07 mV at around 24.544% m_L_. Mix 2 shows a similar trend, with the HCP values decreasing from −173.49 mV at 1.449% m_L_ to −793.07 mV at 22.257% m_L_. Mix 3, which exhibited the lowest corrosion damage, shows HCP values ranging from −173.49 mV at 0.418% m_L_ to −793.07 mV at 17.067% m_L_. These values align with the theoretical understanding that increasingly negative HCP values may be indicative of severe corrosion activity, and reinforce the diagnostic validity of HCP as a field measurement tool.

Second-order polynomial regression models were used to characterize the relationships of HCP and m_L_ for each mix. All regression fits yielded R^2^ values of 0.9999, indicating an almost perfect correlation. This level of fit is rarely achievable in experimental or field data, but this outcome is expected due to the deterministic nature of the COMSOL simulation, which eliminated random variability inherent in field measurements. Nonetheless, the near-perfect fit is meaningful as it demonstrates that the simulated HCP values follow a consistent and mathematically significant trend with m_L_; the results thereby provide strong theoretical support for the prospective capability of HCP measurements to serve as predictors of actual corrosion severity under controlled conditions.

Interestingly, despite the common trend shown by the three mixes, there are notable differences between their presented m_L_ values. For one, Mix 1 showed the most severe corrosion, reaching about 24.544% at −793.07 mV. This observation could be indicative that weaker concrete material properties, such as lower strength or higher porosity due to concrete composition, i.e., Mix 1 had the highest water–cement ratio (0.585) and the lowest design strength (18 MPa) [5], result in a more porous concrete matrix that is more vulnerable to chloride ingress and cracking. The high water–cement ratio may have led to an increased risk of corrosion since it increases the porosity and lessens the density of concrete, allowing corrosive agents to penetrate and reach the embedded rebar, thereby accelerating the corrosion process, which could explain the higher HCP m_L_ values in the relationship. Mix 2, by comparison, reached about 22.257% m_L_ at the same HCP, and the curve showed a slightly more gradual decline compared to that of Mix 1. This suggests that Mix 2 sustained less severe corrosion at comparable HCP values and may be reflective of intermediate concrete quality with more resistance to corrosion. Mix 2 had a more moderate water–cement ratio (0.507) and design strength (24 MPa), providing a better resistance to corrosion if observed from its less severe deterioration trend compared to Mix 1. The m_L_ of Mix 3, which was limited to 17.067%, was the concrete mix that had consistently the least severe mass loss, with the HCP decline being more gradual throughout. Consistent with the previous observations, Mix 3, with the lowest water–cement ratio (0.346) and highest design strength (40 MPa), may have contributed to a denser structure, lower permeability, and greater resistance against corrosion. This may suggest that higher-strength or denser concrete provided better resistance to the progression of corrosion, delaying the onset of the rapid deterioration despite the increasingly negative HCP values. These differences may be attributed to microstructural and mechanical properties of concrete, where stronger mixes generally exhibit lower permeability and fewer pore saturation, which reduces ionic transport and delays the onset of corrosion. In contrast, the weaker mixes may crack earlier and allow aggressive agents to permeate more easily and accelerate corrosion rates. Thus, the comparative analysis posits that concrete mix designs play a role in modulating the progression of corrosion even when the electrochemical measurements, i.e., HCP values in this research, show similar overall trajectories, and are thereby worth further exploration.

Further analysis of the simulation results shows that Mix 3 yielded HCP values above the threshold of values more positive than −200 mV, and had correspondingly low m_L_ of 0.418%, supporting the interpretation of the standard by ASTM C876 that corrosion activity is negligible. However, the observations for Mix 1 and Mix 2 show that the m_L_ were 5.114% and 1.499%, respectively, even though their HCP values also fell within or close to the low probability range for corrosion activity of the ASTM C876 criteria. The difference between 0.418% and 1.499%, much less than 5.114%, is not negligible as generally m_L_ values of less than 1% would be considered to have little adverse effect but m_L_ values between 1% to 10% already have observable effects such as reduced bond strength or structural performance [73,74,75]. This discrepancy shows that HCP is currently used as a probabilistic electrochemical measurement as an NDT, as readings above −200 mV indicate high likelihood of corrosion passivity, but it does not absolutely preclude that there might be localized corrosion, especially when factors such as cracks, chloride ingress, or concrete composition variability are present. It also reinforces that the m_L_ values used in this study were derived from published experimental relationships rather than directly measured in this simulation. As such, the differences between ASTM’s general thresholds and the mix-specific m_L_ outcomes may reflect the unique conditions of those experiments referenced, which lie beyond the scope of the present stationary simulation but nonetheless encourages further investigation. On one hand, the results for Mix 3 align well with the interpretations of the ASTM C876 thresholds, while on the other, Mix 1 demonstrates that HCP readings above −200 mV can still coexist with measurable mass loss under certain conditions.

The average of the relationships of the HCP and m_L_ of the three concrete mixes were taken and fitted into a single regression curve, as shown in Figure 11**,** in order to consolidate and compare the findings from the three different mixes. Although the averaged model was developed from three mixes, its predictive behavior can be generalized because all mixes share similar electrochemical parameters (e.g., corrosion current density range, resistivity spectrum). The averaging process reduces local mix-specific variability, producing a representative correlation applicable to other concrete compositions with comparable mechanical and transport properties. The resulting polynomial best-fit line is

(10) $$\;{HCP}_{average}=1.4697\cdot {m_{L.average}}^{2}-67.186\cdot m_{L.average}-24.967$$

A second-order polynomial provided the best fit balance between simplicity and goodness of fit (R^2^ = 0.9999), as higher-order models showed no significant improvement and risked overfitting. The high coefficient of determination, R^2^ = 0.9999, suggests the strength of the averaged model and its capacity to capture the common trajectory across the different mixes. The averaged curve, shown in Figure 11, confirms the overall nonlinear decline in HCP with the increasing m_L_, showing that the HCP values do not decrease linearly but follow a polynomial curved pattern. Importantly, however, this curve represents an average trend as the three mixes each exhibited distinct variances in their slopes and ranges of m_L_. For instance, Mix 1 demonstrated a steep decline in HCP values with increasing m_L_, while Mix 2 and Mix 3 showed relatively more gradual declines, and these differences may be reflective of the influence of concrete composition quality, e.g., strength, permeability, and water–cement ratio, on the rate of corrosion. The averaged curve and the separate results of the mixes show that the differences between the mixes influence the rate of corrosion despite not affecting the overall trend of the general nonlinear pattern of HCP decline, shown to be consistent across all mixes.

At low levels of m_L_, that are less than 5%, the decline in HCP is shown to be relatively gradual, with the values remaining in the range of about −173 mV to −276 mV. As the m_L_ progresses and reaches the 5% to 10% range, the slope of the curve drops into the −370 mV to −562 mV range. At 15% to 20% m_L_ values, the HCP ranges from −712 mV to −777 mV. Once the m_L_ exceeds around 20%, however, the curve approaches a plateau, with HCP values converging around −780 mV to −793 mV across the mixes, suggesting that at severe corrosion levels, further deterioration in M m_L_ may produce diminishing changes in HCP values.

### 4.3. Validation of Results Through Experimental Investigation

**Figure 12:**
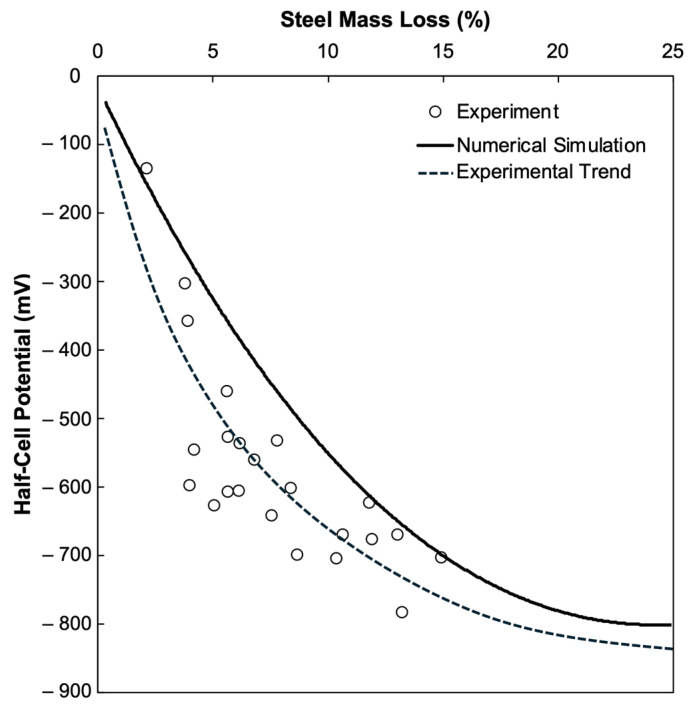
The relationship of steel mass loss (m_L_) and half-cell potential (HCP) from the conducted experiment vs. the COMSOL simulation.

Figure 12 illustrates the comparative relationship between the experimentally measured and numerically simulated HCP values against corresponding steel mass loss. The figure shows both datasets following a distinct nonlinear trend, with negative potentials increasing as corrosion severity rises. Figure 12 demonstrates the relationship of HCP and m_L_ from the experimental investigation in comparison to the results from the numerical simulation. The simulation curve, which shows a distinct nonlinear trend where the HCP becomes more negative as m_L_ increases, generally captures the overall trajectory of the experimental results, although there are observable deviations. The simulated HCP–mass loss trends are consistent with those reported by studies [49], which also observed a nonlinear increase in corrosion potential with rising steel loss. The experimental data show a greater scatter, particularly for 5% to 11% m_L_ where the HCP values range from −400 mV to −700 mV. The approximation curve, less smooth than the simulation curve, demonstrates consistency between the experimental observations and the numerical model while displaying the variability on the measured values. This variability may be expected from field data due to factors such as localized chloride ingress, concrete composition, microcracking, or measurement uncertainties, which were not present in the controlled setup of the numerical simulation. Nonetheless, the general alignment of the experimental data with the simulated HCP values provide support for the capacity of the simulation to approximate the corrosion process under realistic conditions and, subsequently, the HCP readings.

The difference between the curve of the simulated curve and the experimental data also highlights the necessity to capture the variability inherent in field or laboratory measurements in the future development of simulation models for better interpretation as the observed scatter may be attributed to material and environmental complexities that are, for this study, out of the scope of the stationary simulation. The estimated standard deviation of experimental HCP values ranged from ±35 mV to ±70 mV. The main deviations, particularly between −500 mV and −700 mV, stem from intermediate corrosion stages where localized corrosion cells coexist, producing unstable potentials. For a measured HCP of −600 mV, the experimental steel mass loss averaged 4%, while the simulated value was ≈11%. This 63% deviation is attributed to the inherent simplification of the deterministic 2D model used in this numerical simulation as it followed a controlled setup, which assumes uniform corrosion and steady condition, whereas the experimental specimens are influenced by various factors that arise such as moisture condition, cracks, oxygen diffusion, and other environmental factors that are not considered or represented in the numerical model. These environmental and material factors that are inevitably present in an experiment contribute to higher dispersion in the experimental data. Consequently, the simulation tends to slightly overestimate corrosion severity. It is recommended that future studies improve the model by incorporating transient environmental parameters, moisture transport, and others to enhance the predictive realism of the numerical model. The findings, however, reinforce the potential of HCP and numerical models as a tool to predict actual corrosion severity.

### 4.4. Practical Applications and Future Recommendations

It was noted that despite having an overall consistent trend, the magnitude of the m_L_ values displayed differences between the mixes, which highlighted the role of concrete composition, e.g., concrete strength and permeability, as factors affecting the rate of deterioration of the embedded rebar. The findings of this research provide significant implications for furthering NDT methods utilizing HCP measurements and structural health monitoring and maintenance, as it lays the groundwork for developing predictive models that may be used to help correlate non-destructive measurements with actual damage metrics and diagnostic ranges. By incorporating environmental variables, e.g., chloride ingress, carbonation, moisture fluctuations, or temperature, the framework can become an innovative tool for the prediction of the service life of reinforced concrete structures. This research acknowledges that the deterministic simulation environment lacks the variability present in real structures, and future work should therefore attempt to integrate time-dependent diffusion and transport processes, as well as validate the numerical findings against long-term field data. It would make a better conclusion in the future to incorporate multi-physics couplings and mechanics to enhance the predictive accuracy of numerical models.

From the regression of averaging the relationships from the three mixes, observed in Figure 11, Table 6 was derived as a crude but preliminary classification that links specific ranges to approximate m_L_ values. This was made through simple identification of which m_L_ values correspond to typical HCP thresholds within the averaged regression curve, less than −200 mV for light corrosion, around −200 mV to less negative than −500 mV for moderate corrosion, and more negative than −500 mV for severe corrosion. While ASTM C876 traditionally interprets HCP values only in terms of probability of corrosion activity, this classification expands its practical diagnostic potential by relating HCP values with quantifiable m_L_. Such a framework illustrates the prospect of integrating numerical models with NDT techniques and enabling engineers to not only detect the likelihood of corrosion but also estimate the extent of damage in a similar non-destructive manner.

Table 6 demonstrates how numerical modeling may evolve HCP from a probabilistic indicator into a predictive tool for actual steel loss assessment. This enhances its utility as a practical NDT method to support decision-making in reinforced concrete maintenance. A predictive framework using numerical models may be developed for corrosion assessment through connecting specific HCP values with expected steel loss, and this may support engineers and proponents in evaluating reinforced concrete structures while reducing reliance on intrusive or destructive inspections.

For future applications, it is recommended that the developed numerical framework be validated and refined using a broader and more diverse experimental dataset. This should include specimens with varying concrete cover thicknesses, different rebar diameters, levels of concrete saturation, and crack conditions, as these parameters significantly influence the electrochemical response of reinforced concrete. Incorporating these variables will improve the model’s robustness and enable a more accurate representation of real service conditions. Furthermore, long-term monitoring under different environmental exposures would allow for better calibration of the numerical simulations against field behavior. Expanding the dataset and validation scenarios will strengthen the predictive capability of the proposed model, paving the way for its integration into practical corrosion assessment and durability design strategies for existing and new concrete infrastructure.

In addition to expanding the parameter range of the numerical model, future studies are encouraged to explore the combined use of HCP and ER for more accurate prediction of steel mass loss in reinforced concrete. While HCP effectively reflects the electrochemical activity of the steel surface, ER provides valuable information on the concrete’s ionic transport properties, which influence corrosion kinetics [76,77]. Integrating these two non-destructive indicators into a unified predictive framework could reduce uncertainty in corrosion assessment and improve the robustness of predictive models, particularly under variable environmental conditions. Such a combined approach may also facilitate the development of threshold-based deterioration maps and machine learning models for real-time structural health monitoring and durability management.

### 4.5. Discussion

The results discussed in Section 4.1, Section 4.2 and Section 4.3 demonstrate a consistent nonlinear relationship between HCP and mL, supported by both numerical and experimental analyses. The numerical simulation (Section 4.1) captured the electrochemical behavior of corrosion through potential distribution contours that mirrored anodic activity around the rebar. The relationship between HCP, J_(_corr_)_, and re confirmed that higher conductivity and current densities produce more negative potentials, reflecting the expected electrochemical trends in active corrosion systems.

Section 4.2 established a quantitative link between simulated HCP values and corresponding steel mass loss derived from empirical datasets. The regression yielded a second-order polynomial with a coefficient of determination R^2^ = 0.9999, demonstrating a strong fit between predicted and observed values. Across all concrete mixes, increasingly negative HCP corresponded to higher mL values, indicating that HCP can serve as a predictive indicator of corrosion severity when calibrated for concrete resistivity and mix design. Section 4.3 validated these findings experimentally, where the nonlinear trend between HCP and mL remained consistent with the numerical results despite greater scatter due to real-world factors such as moisture variability, cracking, and temperature.

When compared with previous research, the current findings align closely with those who observed both nonlinear correlations between corrosion potential and steel deterioration levels [45,51]. Similarly, as studies have established [5,8,25,50], the results highlighted the influence of concrete resistivity on HCP readings, and confirmed the importance of accounting for the influence of material and environmental factors, such as moisture content, carbonation, or microcrack formation, supporting the conclusion that electrochemical parameters and material heterogeneity jointly affect corrosion interpretation [78]. The simulated potential gradients in this study also agree with the numerical findings, which reported similar spatial potential distributions and polarization saturation at high corrosion densities [55,69]. Collectively, these comparisons reinforce the applicability of the developed HCP–mL correlation while positing the need for future refinements as incorporating environmental coupling, non-uniform corrosion kinetics, and multiphysics modeling can improve the predictive realism and bring numerical simulations closer to field conditions, a potential that can provide quantified classifications and practical applications as those discussed in Section 4.4.

## 5. Conclusions

This study established a quantitative relationship between HCP and steel mass loss in corroded reinforced concrete through combined numerical modeling using COMSOL Multiphysics and experimental validation: The findings demonstrate that HCP measurements can be transformed from simple probabilistic indicators into a practical, predictive tool for evaluating corrosion severity.

1. A clear and strong polynomial relationship was observed between corrosion current density, electrical resistivity, and HCP values, confirming that electrochemical response can be accurately represented through numerical modeling. This study demonstrated an excellent correlation between simulated HCP values and measured steel mass loss, indicating that HCP can be used as a quantitative indicator of corrosion severity when properly calibrated.
2. The correlation between half-cell potential and steel mass loss was best described by a second-order polynomial, ${HCP}_{average}=1.4697\cdot {m_{L.average}}^{2}-67.186\cdot m_{L.average}-24.967$, with R^2^ = 0.9999.
3. HCP measurements obtained at the center of the concrete surface using a PROCEQ Profometer closely matched the numerical predictions, validating the applicability of the model despite minor variability in intermediate corrosion levels.
4. A preliminary HCP–mass loss classification was developed, providing threshold ranges that can support condition-based maintenance strategies and corrosion monitoring in reinforced concrete structures. Future studies should incorporate variables such as concrete cover thickness, rebar diameter, moisture saturation, and crack conditions, supported by a larger and more diverse experimental dataset, to further refine and generalize the predictive framework.

This study is limited by its deterministic 2D steady-state model, which does not capture time-dependent chloride ingress or mechanical cracking effects. Future extensions should incorporate multi-physics coupling of electrochemical, transport, and mechanical phenomena, and validate against long-term field data to enhance generalizability.

## Figures and Tables

**Table 1:** Corrosion activity according to half-cell values [50].

Probability of Steel Corrosion	Half-Cell Potential for Cu/CuSO_4_ Electrode (E_corr_) [mV]
Low (<10%)	*E_corr_* > −200
Intermediate	−200 < *E_corr_* < −350
High (>90%)	*E_corr_* < −350

**Table 2:** Input parameters for J_(_corr_)_ and σ used in COMSOL simulation.

J_(_corr_)_—[A/m^2^]	σ—[S/m]
0.015	0.003554997
0.025	0.003717581
0.035	0.003886186
0.045	0.004068853
0.06	0.004382456
0.075	0.004755785
0.09	0.005196077
0.105	0.005704476
0.115	0.006079472
0.125	0.006480828

**Table 3:** Concrete compositions of Mix 1, Mix 2, and Mix 3.

Properties	Mix 1 (kg/m^3^)	Mix 2 (kg/m^3^)	Mix 3 (kg/m^3^)
Water	168	170	166
Portland Cement Type I	287	335	480
Gravel Sand High-Performance Air-Entraining Agent	898 957 2.58	956 870 2.5	993 720 4.32

**Table 4:** Relationship between m_L_ and *r_e_* per mix, where y is the m_L_ (%) and x is the *r_e_*.

Concrete Mix	Regression	R^2^ Value
Mix 1	$m_{L}=-43.049r_{e}+48.153$	R^2^ = 0.8078
Mix 2	$m_{L}=-46.091r_{e}+47.54$	R^2^ = 0.9781
Mix 3	$m_{L}=-36.879r_{e}+37.297$	R^2^ = 0.9971

**Table 5:** Concrete composition used in the experiment.

Properties	Measurement
Water	168 kg/m^3^
Portland Cement Type I	287 kg/m^3^
Gravel	898 kg/m^3^
Sand	957 kg/m^3^
High-Performance Air-Entraining Agent	2.58 kg/m^3^
Design Strength	18 MPa
W/c Ratio	0.585

**Table 6:** Preliminary classification of half-cell potential and steel mass loss (HCP- m_L_) quantified.

HCP	m_L_	Description
≤−200 mV	~0–2%	Light corrosion
−200 mV < x < −500 mV	~2–7%	Moderate corrosion
≥−500 mV	~7–16%	Severe corrosion

## Data Availability

The data presented in this study are available from the corresponding author upon reasonable request. However, the dataset cannot be made publicly available at this time because it forms part of a larger, ongoing research project.

## References

[1] Valipour M., Yekkalar M., Shekarchi M., Panahi S. (2014). Environmental Assessment of Green Concrete Containing Natural Zeolite on the Global Warming Index in Marine Environments. J. Clean. Prod..

[2] Kubba S. (2017). Green Building Materials and Products. Handbook of Green Building Design and Construction.

[3] Zeng H., Qu S., Tian Y., Hu Y., Li Y. (2023). Recent Progress on Graphene Oxide for Next-Generation Concrete: Characterizations, Applications and Challenges. J. Build. Eng..

[4] Mukhti J.A., Robles K.P.V., Lee K.-H., Kee S.-H., Mukhti J.A., Paolo K., Robles V., Lee K.-H., Kee S.-H. (2023). Evaluation of Early Concrete Damage Caused by Chloride-Induced Steel Corrosion Using a Deep Learning Approach Based on RNN for Ultrasonic Pulse Waves. Materials.

[5] Robles K.P.V., Gucunski N., Kee S.H. (2024). Evaluation of Steel Corrosion-Induced Concrete Damage Using Electrical Resistivity Measurements. Constr. Build. Mater..

[6] Li S. (2021). Evaluation of Ecological Environmental Pollution in Green Building Construction. Nat. Environ. Pollut. Technol..

[7] Liang Y., Wang L. (2020). Prediction of Corrosion-Induced Cracking of Concrete Cover: A Critical Review for Thick-Walled Cylinder Models. Ocean Eng..

[8] Song H.-W., Saraswathy V. (2007). Corrosion Monitoring of Reinforced Concrete Structures—A Review. Int. J. Electrochem. Sci..

[9] Yodsudjai W., Pattarakittam T. (2017). Factors Influencing Half-Cell Potential Measurement and Its Relationship with Corrosion Level. Measurement.

[10] Leelalerkiet V., Kyung J.-W., Ohtsu M., Yokota M. (2004). Analysis of Half-Cell Potential Measurement for Corrosion of Reinforced Concrete. Constr. Build. Mater..

[11] Sohail M.G., Kahraman R., Alnuaimi N.A., Gencturk B., Alnahhal W., Dawood M., Belarbi A. (2020). Electrochemical Behavior of Mild and Corrosion Resistant Concrete Reinforcing Steels. Constr. Build. Mater..

[12] Hou B., Li X., Ma X., Du C., Zhang D., Zheng M., Xu W., Lu D., Ma F. (2017). The Cost of Corrosion in China. npj Mater. Degrad..

[13] Dunn R.C., Ross R.A., Davis G.D. Corrosion Monitoring of Steel Reinforced Concrete Structures Using Embedded Instrumentation. Proceedings of the NACE–International Corrosion Conference Series.

[14] Koch G. (2017). Cost of Corrosion. Trends in Oil and Gas Corrosion Research and Technologies.

[15] Papavinasam S., Rebak R.B., Yang L., Berke N.S. (2019). Front Matter. Advances in Electrochemical Techniques for Corrosion Monitoring and Laboratory Corrosion Measurements.

[16] Assessment of the Global Cost of Corrosion. http://impact.nace.org/economic-impact.aspx.

[17] Burkert A., Ebell G., Eichler T., Hariri K., Harnisch J., Keßler S., Mayer T., Meier J., Mietz J., Reichling K. (2014). Electrochemical Half-Cell Potential Measurements for the Detection of Reinforcement Corrosion.

[18] Ali M., Shams M.A., Bheel N., Almaliki A.H., Mahmoud A.S., Dodo Y.A., Benjeddou O. (2024). A Review on Chloride Induced Corrosion in Reinforced Concrete Structures: Lab and In Situ Investigation. RSC Adv..

[19] Sagüés A.A., Sánchez A.N., Lau K., Kranc S.C. (2014). Service Life Forecasting for Reinforced Concrete Incorporating Potential-Dependent Chloride Threshold. Corrosion.

[20] Hover K.C. (2011). The Influence of Water on the Performance of Concrete. Constr. Build. Mater..

[21] Assouli B., Ballivy G., Rivard P. (2008). Influence of Environmental Parameters on Application of Standard ASTM C876-91: Half Cell Potential Measurements. Corros. Eng. Sci. Technol..

[22] Bartholomew J., Bennett J., Turk T., Hartt W.H., Lankard D.R., Sagues A.A., Savinell R. (1993). Control Criteria and Materials Performance Studies for Cathodic Protection of Reinforced Concrete.

[23] Kessler S., Gehlen C. (2016). Influence of Concrete Moisture Condition on Half-Cell Potential Measurement. Proceedings of the 5th International Conference on the Durability of Concrete Structures.

[24] Chen F., Baji H., Li C.Q. (2018). A Comparative Study on Factors Affecting Time to Cover Cracking as a Service Life Indicator. Constr. Build. Mater..

[25] Alexander M., Beushausen H. (2019). Durability, Service Life Prediction, and Modelling for Reinforced Concrete Structures—Review and Critique. Cem. Concr. Res..

[26] Bezuidenhout S.R., van Zijl G.P.A.G. (2019). Corrosion Propagation in Cracked Reinforced Concrete, toward Determining Residual Service Life. Struct. Concr..

[27] Arachchige L.J., Li C., Wang F. (2025). Recent Advances in Understanding Iron/Steel Corrosion: Mechanistic Insights from Molecular Simulations. Curr. Opin. Solid State Mater. Sci..

[28] Robles K.P., Yee J.J., Gucunski N., Kee S.H. (2025). Hybrid Data-Driven Machine Learning Approach for Evaluating Steel Corrosion in Concrete Using Electrical Resistivity and Documented Concrete Performance Indicators. Constr. Build. Mater..

[29] Ahmad S. (2009). Techniques for Inducing Accelerated Corrosion of Steel in Concrete. Arab. J. Sci. Eng..

[30] Ramezanianpour A.A., Pilvar A., Mahdikhani M., Moodi F. (2011). Practical Evaluation of Relationship between Concrete Resistivity, Water Penetration, Rapid Chloride Penetration and Compressive Strength. Constr. Build. Mater..

[31] Ormellese M., Berra M., Bolzoni F., Pastore T. (2006). Corrosion Inhibitors for Chlorides Induced Corrosion in Reinforced Concrete Structures. Cem. Concr. Res..

[32] Angst U.M. (2018). Challenges and Opportunities in Corrosion of Steel in Concrete. Mater. Struct..

[33] Elsener B., Rossi A. (2018). Passivation of Steel and Stainless Steel in Alkaline Media Simulating Concrete. Encyclopedia of Interfacial Chemistry.

[34] Ahmad Z. (2006). Basic Concepts in Corrosion. Principles of Corrosion Engineering and Corrosion Control.

[35] Mei K., He Z., Yi B., Lin X., Wang J., Wang H., Liu J. (2022). Study on Electrochemical Characteristics of Reinforced Concrete Corrosion under the Action of Carbonation and Chloride. Case Stud. Constr. Mater..

[36] Shi X., Xie N., Fortune K., Gong J. (2012). Durability of Steel Reinforced Concrete in Chloride Environments: An Overview. Constr. Build. Mater..

[37] Sadowski L. (2013). Methodology for Assessing the Probability of Corrosion in Concrete Structures on the Basis of Half-Cell Potential and Concrete Resistivity Measurements. Sci. World J..

[38] Law D.W., Cairns J.H. Evaluation of Corrosion Loss of Steel Reinforcing Bars in Concrete Using Linear Polarisation Resistance Measurements. https://www.ndt.net/article/ndtce03/papers/p015/p015.htm.

[39] Moreno M., Morris W., Alvarez M.G., Duffó G.S. (2004). Corrosion of Reinforcing Steel in Simulated Concrete Pore Solutions Effect of Carbonation and Chloride Content. Corros. Sci..

[40] Avila-Mendoza J., Flores J.M., Castillo U.C. (1994). Effect of Superficial Oxides on Corrosion of Steel Reinforcement Embedded in Concrete. Corrosion.

[41] Huet B., L’Hostis V., Miserque F., Idrissi H. (2005). Electrochemical Behavior of Mild Steel in Concrete: Influence of PH and Carbonate Content of Concrete Pore Solution. Electrochim. Acta.

[42] Gonzalez J.A., Andrade C. (1982). Eftect of Carbonation, Chlorides and Relative Ambient Humidity on the Corrosion of Galvanized Rebarsembedded in Concrete. Br. Corros. J..

[43] Hou J., Chung D.D.L. (2000). Effect of Admixtures in Concrete on the Corrosion Resistance of Steel Reinforced Concrete. Corros. Sci..

[44] Robles K.P.V., Yee J.J., Kee S.H. (2022). Electrical Resistivity Measurements for Nondestructive Evaluation of Chloride-Induced Deterioration of Reinforced Concrete—A Review. Materials.

[45] Robles K.P., Kee S.-H. (2022). An Electrical Resistivity Measurement Approach in the Determination of the Degree of Water Saturation of Reinforced Concrete by Machine Learning Methods. J. Acad. Present. Conf. Archit. Inst. Korea.

[46] Sarma C.B., Vartika, Dwivedi S.K., Raja M., Vidyarthi U.S. (2024). Evaluation of the Corrosion Activity of Reinforcement in Drainage Gallery of Concrete Dam Using Half-Cell Potential (HCP). Int. J. Multidiscip. Res..

[47] De Carufel S. (2023). Half-Cell Potential Test: Measurement and Devices. https://www.giatecscientific.com/education/what-is-the-half-cell-potential-test/.

[48] Torres-Acosta A.A. (2024). *Technical Note*: Considerations to Avoid Corrosion Rate Estimate Error of the Reinforcing Steel If Based Only on Concrete’s Electrical Resistivity. Corrosion.

[49] Mohammad P.-G., Isgor O.B., Pouria G. (2009). Quantitative Interpretation of Half-Cell Potential Measurements in Concrete Structures. J. Mater. Civ. Eng..

[50] (2015). Standard Test Method for Corrosion Potentials of Uncoated Reinforcing Steel in Concrete.

[51] Capasso M., Carusi V., Forte A., Lavorato D., Raoli G., Santini S. (2024). A Probabilistic Interpretation of Corrosion State through Half-Cell Potential and Electrical Resistivity Measures: The Flaminio Bridge in Rome. Case Stud. Constr. Mater..

[52] Pfändler P., Keßler S., Huber M., Angst U. (2024). Spatial Variability of Half-Cell Potential Data from a Reinforced Concrete Structure—A Geostatistical Analysis. Struct. Infrastruct. Eng..

[53] Akoba B., Ajah U.C., Kennedy C. (2024). Utilizing Electrochemical Techniques for Assessing the Probability of Concrete Resistivity and Corrosion Potential in Reinforced Concrete Structures. Middle East Res. J. Eng. Technol..

[54] Golam M.A. (1990). Reference Half Cells for Monitoring Corrosion Condition of Steel in Reinforced Concrete Structures. Anti-Corros. Methods Mater..

[55] Elsener B. (2002). Macrocell Corrosion of Steel in Concrete—Implications for Corrosion Monitoring. Cem. Concr. Compos..

[56] Polder R.B. (2001). Test Methods for on Site Measurement of Resistivity of Concrete—A RILEM TC-154 Technical Recommendation. Constr. Build. Mater..

[57] Adriman R., Ibrahim I.B.M., Huzni S., Fonna S., Ariffin A.K. (2022). Improving Half-Cell Potential Survey through Computational Inverse Analysis for Quantitative Corrosion Profiling. Case Stud. Constr. Mater..

[58] Kim K.B., Park K.T., Kwon S.J. (2013). Evaluation of Half Cell Potential Measurement in Cracked Concrete Exposed to Salt Spraying Test. J. Korea Concr. Inst..

[59] Abouhussien A.A., Hassan A.A.A. (2018). Acoustic Emission Monitoring of Corrosion Damage Propagation in Large-Scale Reinforced Concrete Beams. J. Perform. Constr. Facil..

[60] Frølund T., Klinghoffer O., Sørensen H.E. Pro’s and Con’s of Half-Cell Potentials and Corrosion Rate Measurements. Proceedings of the International Conference on Structural Faults + Repairs.

[61] Almashakbeh Y., Saleh E., Al-Akhras N.M. (2022). Evaluation of Half-Cell Potential Measurements for Reinforced Concrete Corrosion. Coatings.

[62] Filipek R., Szyszkiewicz-Warzecha K., Szczudło J. (2020). Corrosion of Steel in Concrete—Modeling of Electrochemical Potential Measurement in 3D Geometry. Arch. Metall. Mater..

[63] (2018). Comsol Multiphysics COMSOL AC/DC Module User’s Guide. https://doc.comsol.com/5.4/doc/com.comsol.help.acdc/ACDCModuleUsersGuide.pdf.

[64] Shevtsov D.S., Zartsyn I.D., Komarova E.S. (2021). Relation between Resistivity of Concrete and Corrosion Rate of Reinforcing Bars Caused by Galvanic Cells in the Presence of Chloride. Cem. Concr. Compos..

[65] Robles K.P.V., Kim D.-W., Kee S.-H., Yee J.-J., Paolo K., Robles V., Kim D.-W., Yee J.-J., Lee J.-W., Kee S.-H. (2020). Electrical Resistivity Measurements of Reinforced Concrete Slabs with Delamination Defects. Sensors.

[66] Robles K.P.V., Yee J.J., Gucunski N., Kee S.H. (2025). Calibrating Electrical Resistivity Measurements in Reinforced Concrete with Rebar Effects and Practical Guidelines. Measurement.

[67] Papakonstantinou C.G., Balaguru P.N., Auyeung Y. (2011). Influence of FRP Confinement on Bond Behavior of Corroded Steel Reinforcement. Cem. Concr. Compos..

[68] Li C., Chen Q., Wang R., Wu M., Jiang Z. (2020). Corrosion Assessment of Reinforced Concrete Structures Exposed to Chloride Environments in Underground Tunnels: Theoretical Insights and Practical Data Interpretations. Cem. Concr. Compos..

[69] Zou Z.H., Wu J., Wang Z., Wang Z. (2016). Relationship between Half-Cell Potential and Corrosion Level of Rebar in Concrete. Corros. Eng. Sci. Technol..

[70] Pakrashi V., Kelly J., O’Connor A., Biondini F., Frangopol D.M. (2012). Direct and Probabilistic Interrelationships between Half-Cell Potential and Resistivity Test Results for Durability Ranking. Bridge Maintenance, Safety, Management, Resilience and Sustainability: Proceedings of the Sixth International Conference on Bridge Maintenance, Safety and Management, Stresa, Italy, 8–12 July 2012.

[71] Lai W.-L., Kind T., Stoppel M., Wiggenhauser H. (2013). Measurement of Accelerated Steel Corrosion in Concrete Using Ground-Penetrating Radar and a Modified Half-Cell Potential Method. J. Infrastruct. Syst..

[72] Wilson J., Yu T. (2013). Accelerated Artificial Corrosion Monitoring of Reinforced Concrete Slabs Using the Half-Cell Potential Method. Proceedings of the Symposium on the Application of Geophysics to Engineering and Environmental Problems.

[73] Kim K.B., Park K.-T., Kwon S.-J. (2013). Variation of Half Cell Potential Measurement in Concrete with Different Properties and Anti-Corrosive Condition. J. Korea Inst. Struct. Maint. Insp..

[74] Giri S. (2021). The Study of Half-Cell Potential Behaviour of Reinforced Concrete in Marine Environment. Int. J. Res. Appl. Sci. Eng. Technol..

[75] Malumbela G., Moyo P., Alexander M. (2011). Influence of Corrosion Crack Patterns on the Rate of Crack Widening of RC Beams. Constr. Build. Mater..

[76] Robles K.P., Kee S.-H. (2022). Experimental Investigation on the Influence of Degree of Saturation to the Effect of Steel Reinforcements to the Electrical Resistivity of Reinforced Concrete. J. Acad. Conf. Korean Concr. Inst..

[77] Robles K.P.V., Yee J.J., Kee S.H. Simulation-Based Assessment of the Impact of Internal and Surface-Breaking Cracks on Reinforced Concrete Electrical Resistivity. Proceedings of the 7th International Conference on Civil Engineering and Architecture.

[78] Banar R., Moodi F., Ramezanianpour A.A., Ramezanianpour A.M., Dashti P. (2025). Experimental and Numerical Simulation of Carbonation-Induced Corrosion in Reinforced Concretes. Case Stud. Constr. Mater..

